# THE USE OF AMBULATORY VERSUS EMERGENCY SERVICES AMONG SENIOR ADULTS WITH LIMITED ENGLISH PROFICIENCY

**DOI:** 10.1093/geroni/igad104.1316

**Published:** 2023-12-21

**Authors:** Allen Glicksman, Adam Davey, Steven Albert, Ari Friedman, Philip Lai, Lauren Ring, Misha Rodriguez, Michael Liebman

**Affiliations:** NewCourtland, Haverford, Pennsylvania, United States; University of Delaware, Newark, Delaware, United States; University of Pittsburgh, Pittsburgh, Pennsylvania, United States; University of Pennsylvania, Philadelphia, Pennsylvania, United States; Philadelphia Senior Center, Philadelphia, Pennsylvania, United States; Private, Philadelphia, Pennsylvania, United States; APM, Philadelphia, Pennsylvania, United States; IPQ Analytics Inc, Kennett Square, Pennsylvania, United States

## Abstract

Senior adults who are immigrants with limited English proficiency may have difficulty accessing health and social services. However, identifying patients with LEP in large databases such as insurance claims has historically been challenging. We overcome this with two strategies. First, we conducted focus groups among older Spanish and Chinese speakers. Based on the findings we hypothesize that 1) we can expect distinct patterns of service use associated with a common language and 2) Chinese speakers are more likely than Spanish speakers to use ambulatory services and less likely to use emergency services because Chinese speakers feel more confident in their ability to navigate formal systems. Next, we obtained data on Spanish and Chinese speakers aged 60 and older and living in Philadelphia from a regional health information exchange, including routinely collected data on patients’ primary language and English proficiency. We are using the data to test our hypotheses regarding these specific LEP populations. The data will be used as pilot for a study to further investigate attitudes and perceptions of formal care providers in these two LEP communities.

